# Supplementary material for: A Novel Near-Infrared Ytterbium Complex [Yb(DPPDA)2](DIPEA) with Φ = 0.46% and τobs = 105 μs
Source: Molecules. 2023 Feb 8;28(4):1632. doi: 10.3390/molecules28041632 (PMC9965908; doi:10.3390/molecules28041632)
Supplement: Supplementary file 1 [file molecules-28-01632-s001.zip › molecules-2089122-supplementary.pdf]

molecules

MDPI

Supporting information

A novel near-infrared ytterbium complex $[Yb(DPPDA)_2](DIPEA)$ with φ = 0.46% and τ_{obs} = 105 μs

Guozhu Ren [1,2], Danyang Zhang [1,2], Hao Wang [3], Xiaofang Li [1,2], Ruiping Deng [1], Shihong Zhou [1], Long Tian [1], Liang Zhou [1,2,]*

1 State Key Laboratory of Rare Earth Resource Utilization, Changchun Institute of Applied Chemistry,
Chinese Academy of Sciences, Changchun 130022, China; rgz666@ciac.ac.cn (G.R.);
dyzhang26@ciac.ac.cn (D.Z.); xflizkd@ciac.ac.cn (X.L.); dengrp@ciac.ac.cn (R.D.); shzhou@ciac.ac.cn (S.Z.); tianlong@ciac.ac.cn (L.T.)
2 School of Applied Chemistry and Engineering, University of Science and Technology of China,
Hefei 230027, China
3 School of Materials Science and Engineering, Jilin Jianzhu University, Changchun 130118, China;
13341427152@163.com
* Correspondence: zhoul@ciac.ac.cn; Tel.: +86-431-85262855; Fax: +86-431-85698041

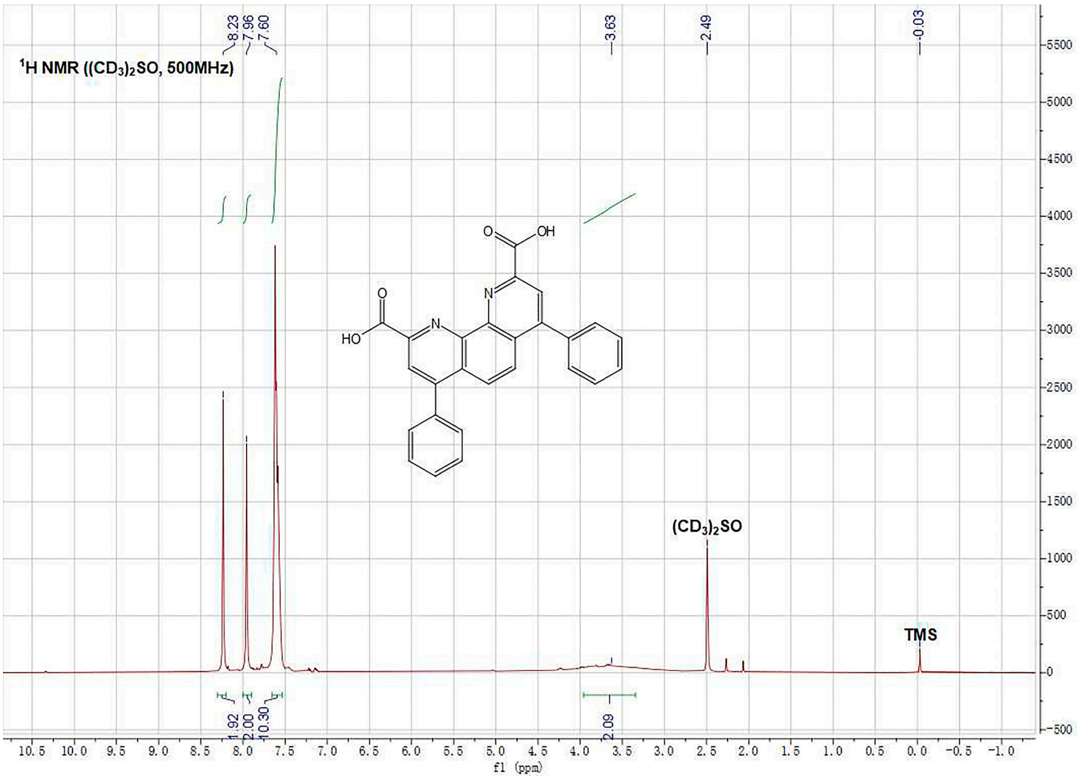

Figure S1. ^{1}H NMR spectrum of 4,7-diphenyl-1,10-phenanthroline-2,9-dicarboxylic acid (DPPDA).

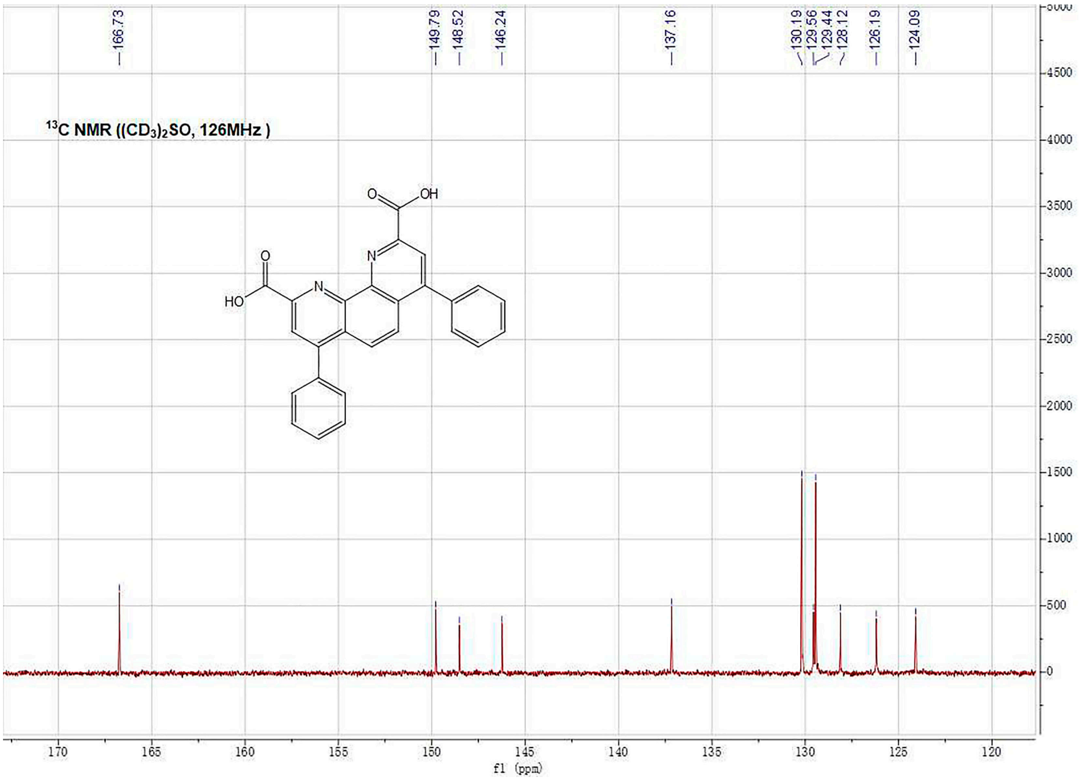

Figure S2. ^{13}C NMR spectrum of DPPDA.

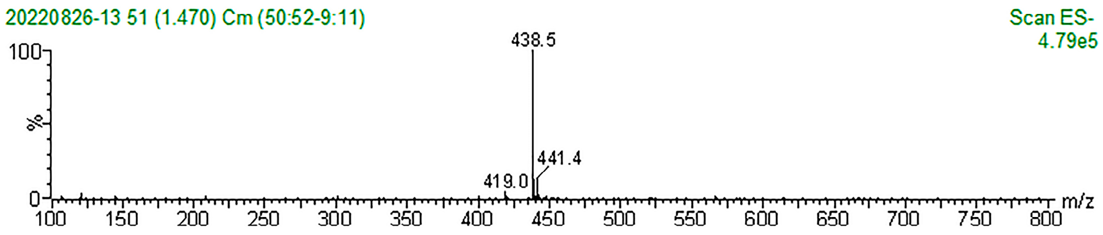

Figure S3. ESI-MS spectrum of DPPDA (CH_3CH_2OH negative mode).

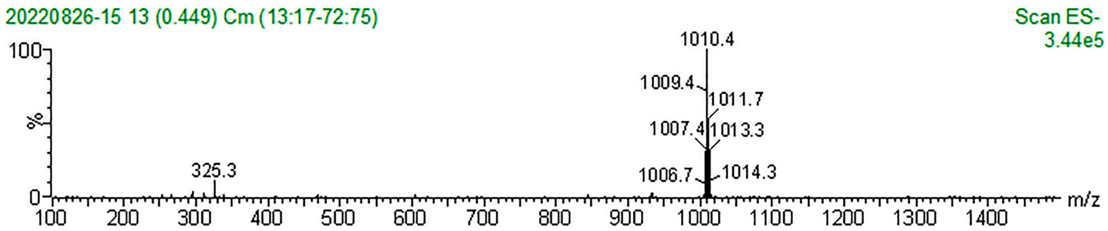

Figure S4. ESI-MS spectrum of [Yb(DPPDA)$_2$](DIPEA) ($CHCl_3$ negative mode).

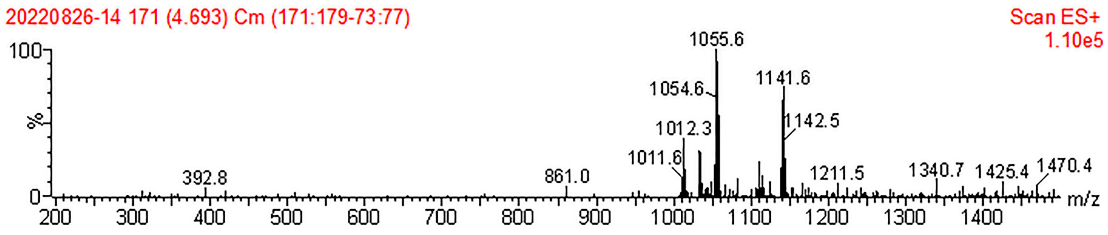

molecules

MDPI

Figure S5. ESI-MS spectrum of [Yb(DPPDA)2](DIPEA) (CHCl3 positive mode).

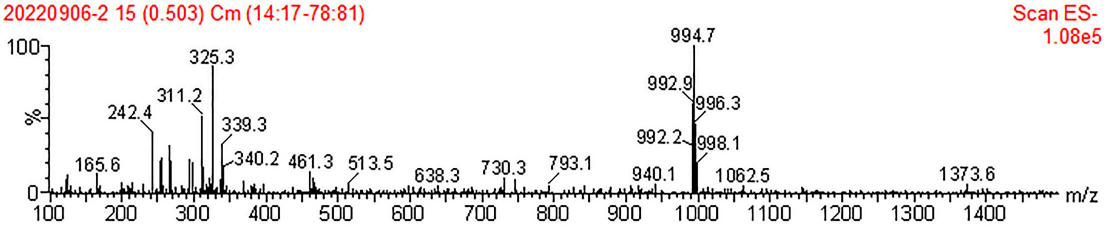

Figure S6. ESI-MS spectrum of [Gd(DPPDA)2](DIPEA) (CHCl3 negative mode).

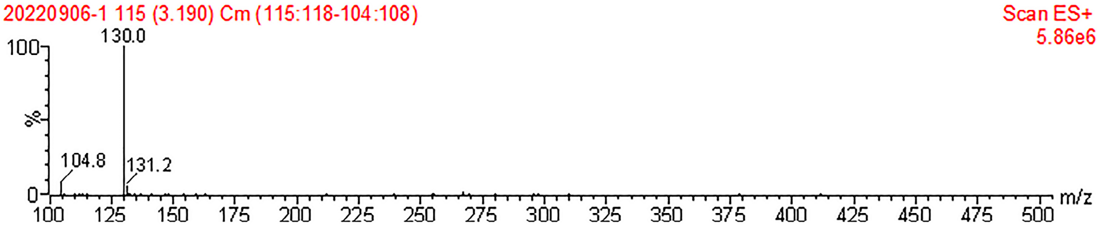

Figure S7. ESI-MS spectrum of [Gd(DPPDA)2](DIPEA) (CHCl3 positive mode).

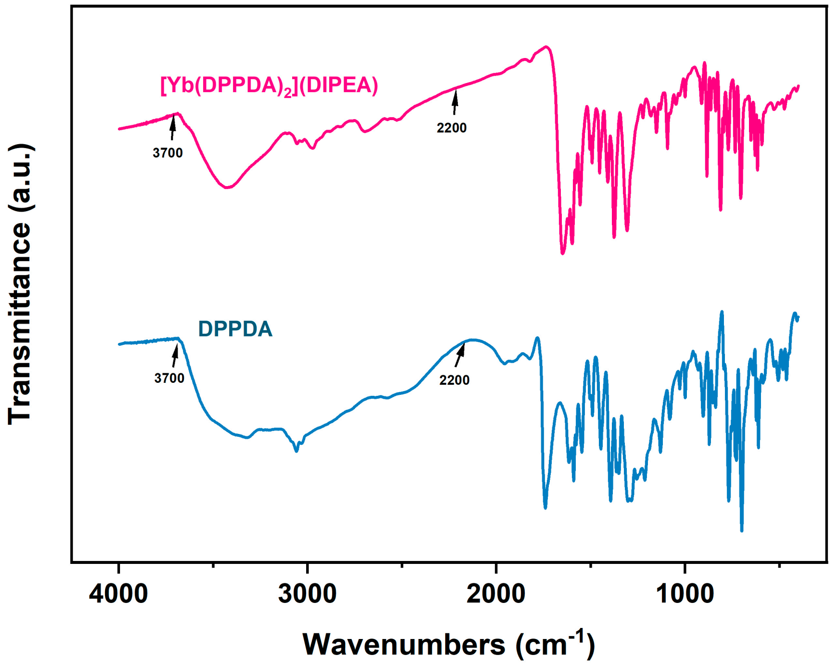

Figure S8. FT-IR spectra of DPPDA and [Yb(DPPDA)2](DIPEA) in the range of 4000~400 cm^{-1}.

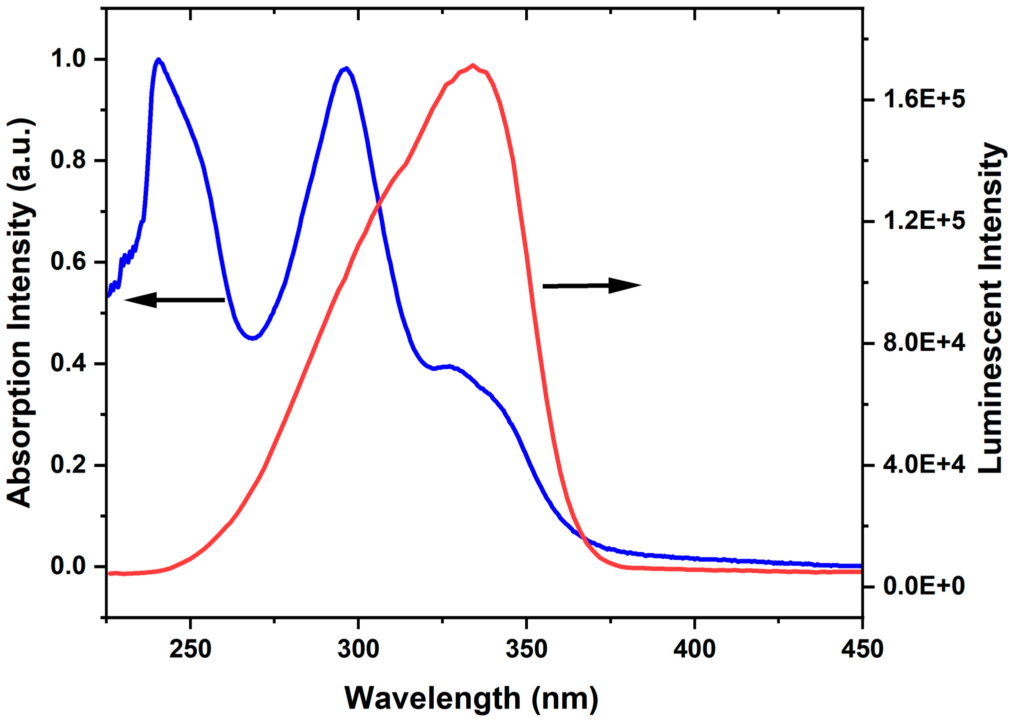

Figure S9. UV-Vis absorption spectrum and excitation spectrum ($\lambda_{monitor}$ = 1011 nm) of $[Yb(DPPDA)_2](DIPEA)$ at room temperature.

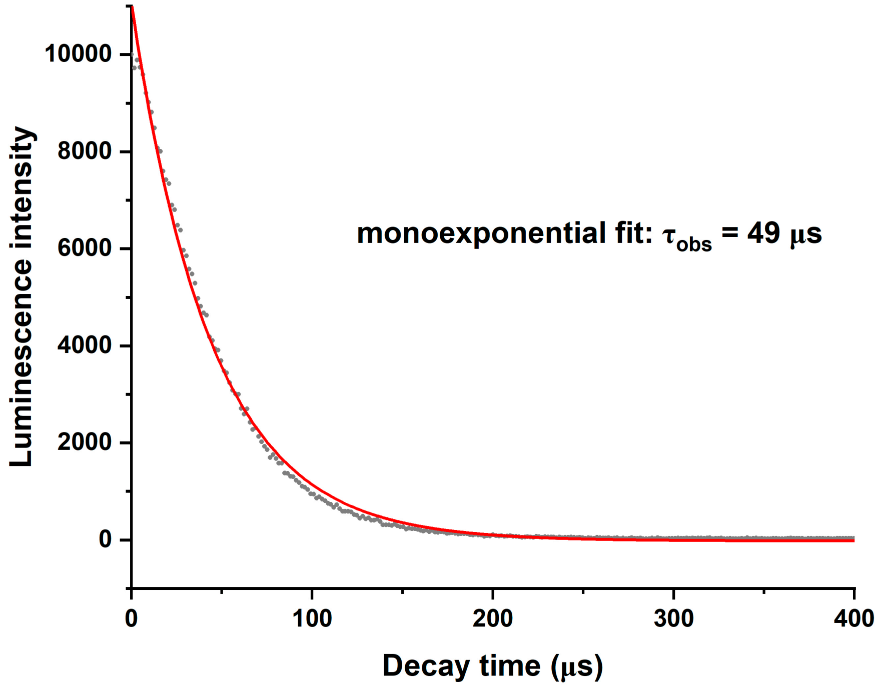

molecules
MDPI

Figure S10. Luminescence decay profile of the transition $^2F_{5/2}\rightarrow{}^2F_{7/2}$ (λ_{em} = 1011 nm) in $[Yb(DPPDA)_2](DIPEA)$ ($CHCl_3$, λ_{ex} = 335 nm, monoexponential fit in red) at room temperature.

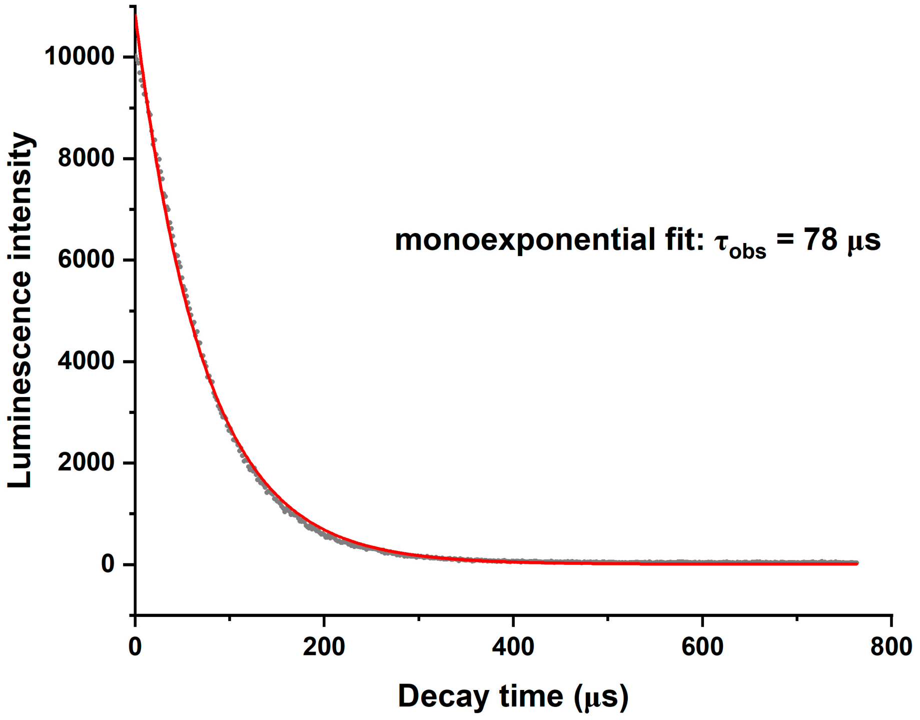

Figure S11. Luminescence decay profile of the transition $^2F_{5/2}\rightarrow{}^2F_{7/2}$ (λ_{em} = 1011 nm) in $[Yb(DPPDA)_2](DIPEA)$ ($CDCl_3$, λ_{ex} = 335 nm, monoexponential fit in red) at room temperature.

molecules MDPI

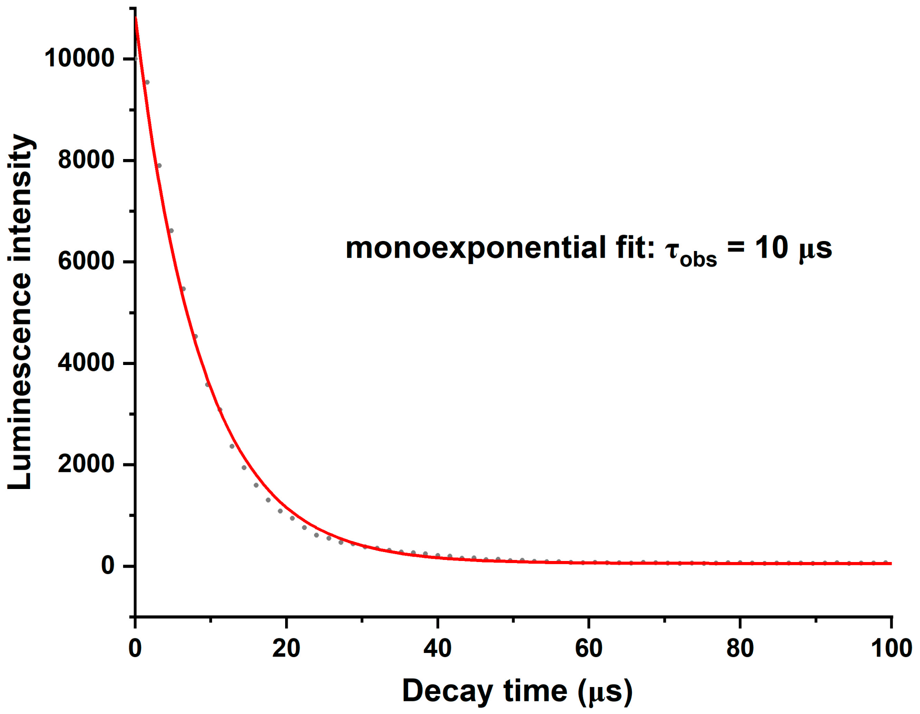

Figure S12. Luminescence decay profile of the transition $^2F_{5/2}$→$^2F_{7/2}$ (λ_{em} = 1011 nm) in [Yb(DPPDA)$_2$](DIPEA) (CH_3OH, λ_{ex} = 335 nm, monoexponential fit in red) at room temperature.

molecules

MDPI

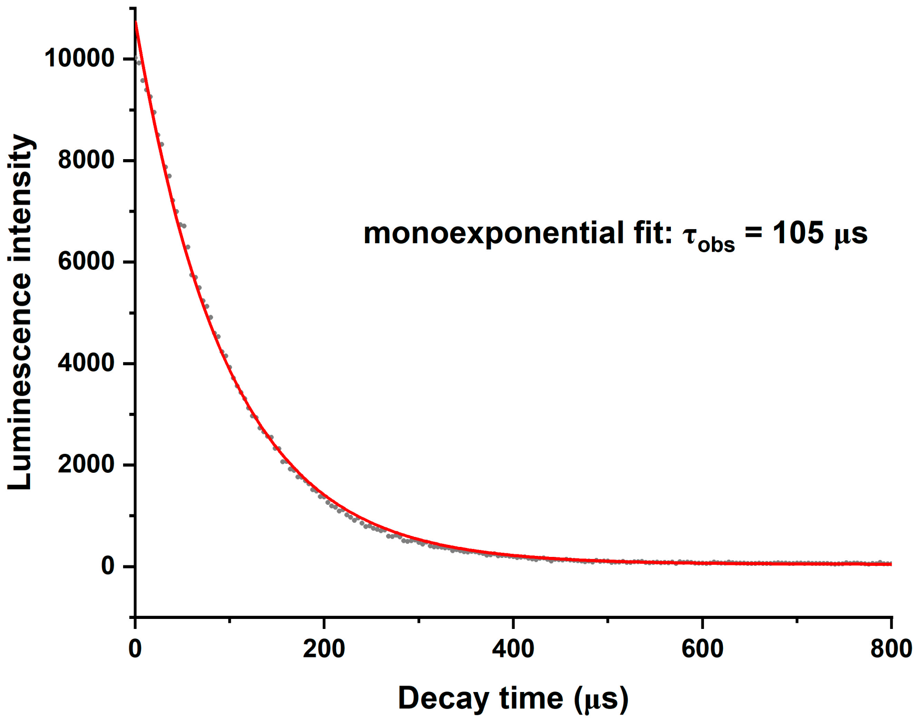

Figure S13. Luminescence decay profile of the transition $^2F_{5/2} \rightarrow {}^2F_{7/2}$ (λ_{em} = 1011 nm) in [Yb(DPPDA)$_2$](DIPEA) (CD_3OD, λ_{ex} = 335 nm, monoexponential fit in red) at room temperature.

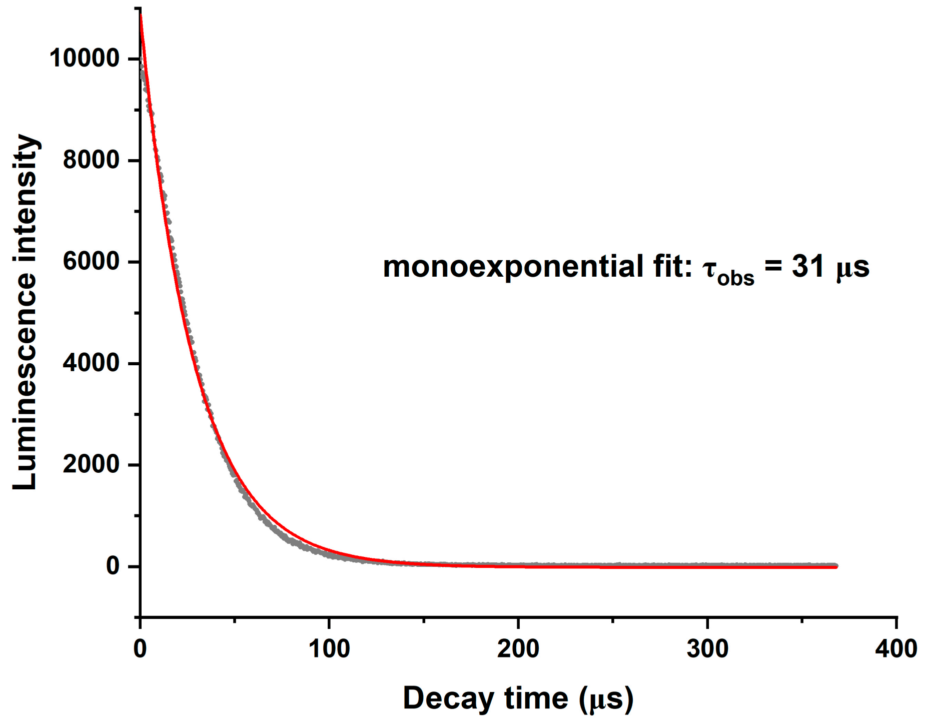

Figure S14. Luminescence decay profile of the transition $^2F_{5/2}$→$^2F_{7/2}$ (λ_{em} = 1011 nm) in [Yb(DPPDA)$_2$](DIPEA) (solid, λ_{ex} = 335 nm, monoexponential fit in red) at room temperature.

Solvent[1]	S_R[2]	S_S[3]	E_S[4]	QYs[5] (%)
solid	9.26E+08	8.10E+07	1.31E+05	0.06
CH_3OH	1.04E+09	3.18E+07	3.98E+04	0.02
CD_3OD	4.95E+08	1.56E+07	5.58E+05	0.46
$CHCl_3$	4.89E+08	1.66E+07	2.69E+05	0.22
$CDCl_3$	4.31E+08	2.63E+07	3.87E+05	0.37

[1]Measuring condition: λ_{ex} = 335 nm, λ_{em} = 1011 nm, 1×10^{-4} mol/L, 298 K. [2]S_R = Integrated scattering intensity of reference cell. [3]S_S = Integrated scattering intensity of sample. [4]E_S = Integrated emission intensity of sample. [5]The calculation formula of QYs is: (F*E_S)/(S_R-S_S), F is correction factor aimed to eliminate the influence of different sensitivity between UV-Vis detector (180 nm—900 nm) and NIR detector (600 nm—1700 nm). By measuring and contrasting detected light intensity of the spectral overlapping region (700 nm—800 nm) of two detectors, F is calculated to be 3.91.

Table S1. The measuring data of absolute quantum yields of [Yb(DPPDA)$_2$](DIPEA) in different solutions and solid state at room temperature.

molecules

MDPI

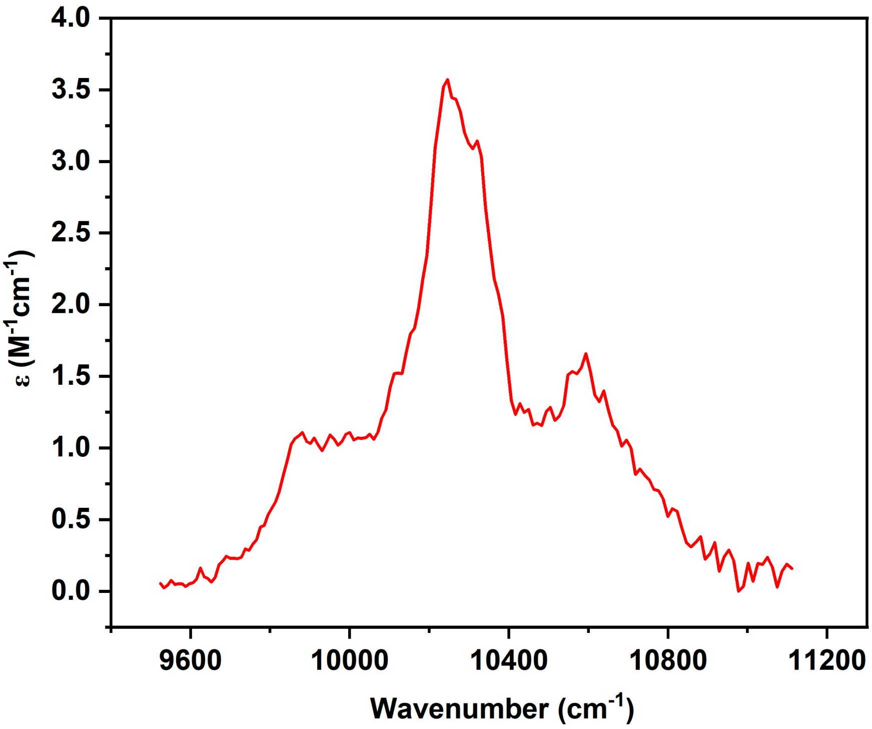

Figure S15. NIR absorption spectrum of the f-f transition $^2F_{7/2} \rightarrow ^2F_{5/2}$ in $[Yb(DPPDA)_2](DIPEA)$ (in 4×10^{-4} mol/L CD_3OD solution).

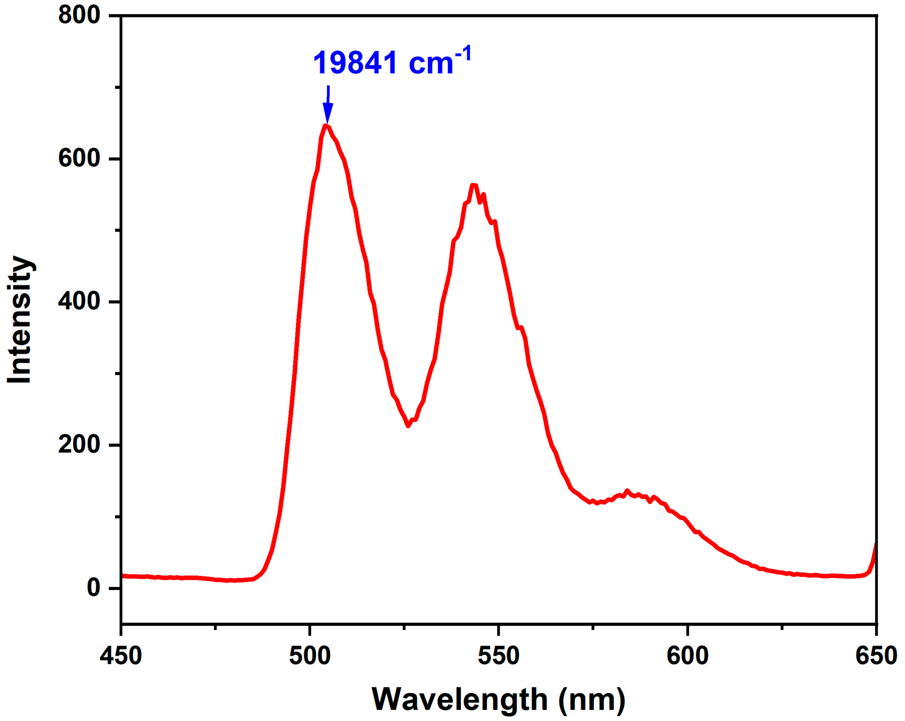

Figure S16. Low temperature fluorescence spectrum of [Gd(DPPDA)$_2$](DIPEA) (λ_{ex} = 335 nm, in 1×10^{-4} mol/L $CHCl_3$ solution).

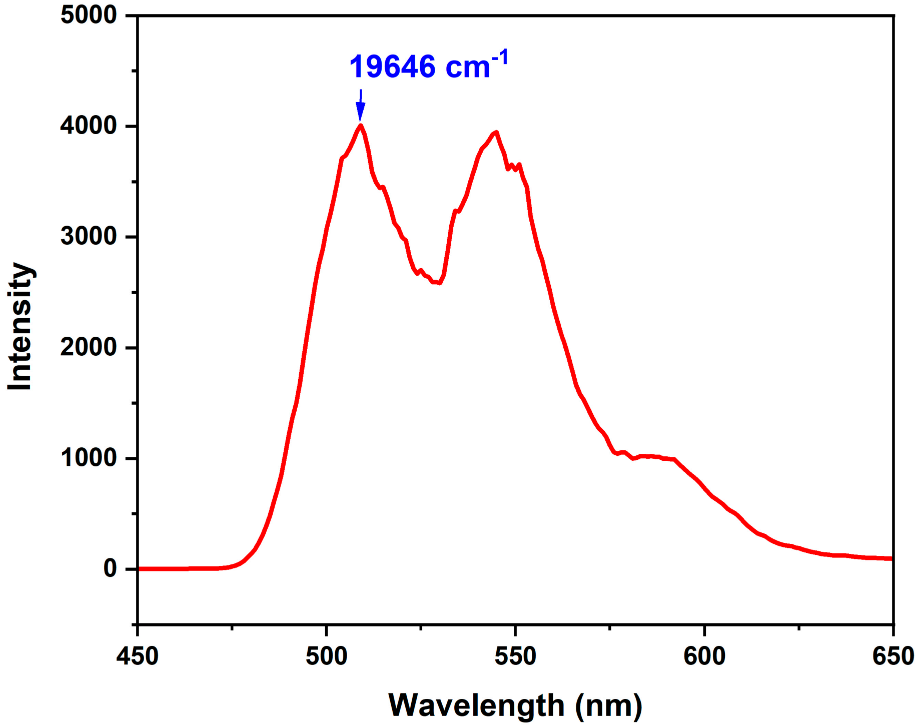

molecules

MDPI

Figure S17. Low temperature phosphorescence spectrum of [Gd(DPPDA)$_2$](DIPEA) (λ_{ex} = 335 nm, in 1×10^{-4} mol/L $CHCl_3$ solution).

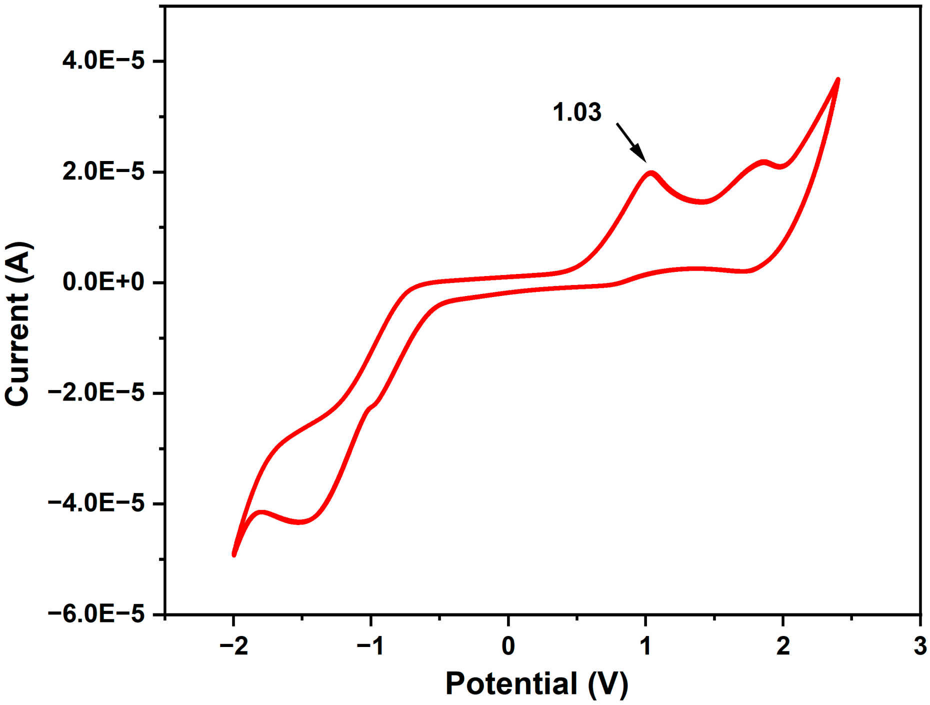

Figure S18. Cyclic voltammogram of DPPDA(DIPEA)$_2$ (with Ag/AgCl reference electrode in 1×10^{-3} mol/L $CHCl_3$ solution).

molecules

MDPI

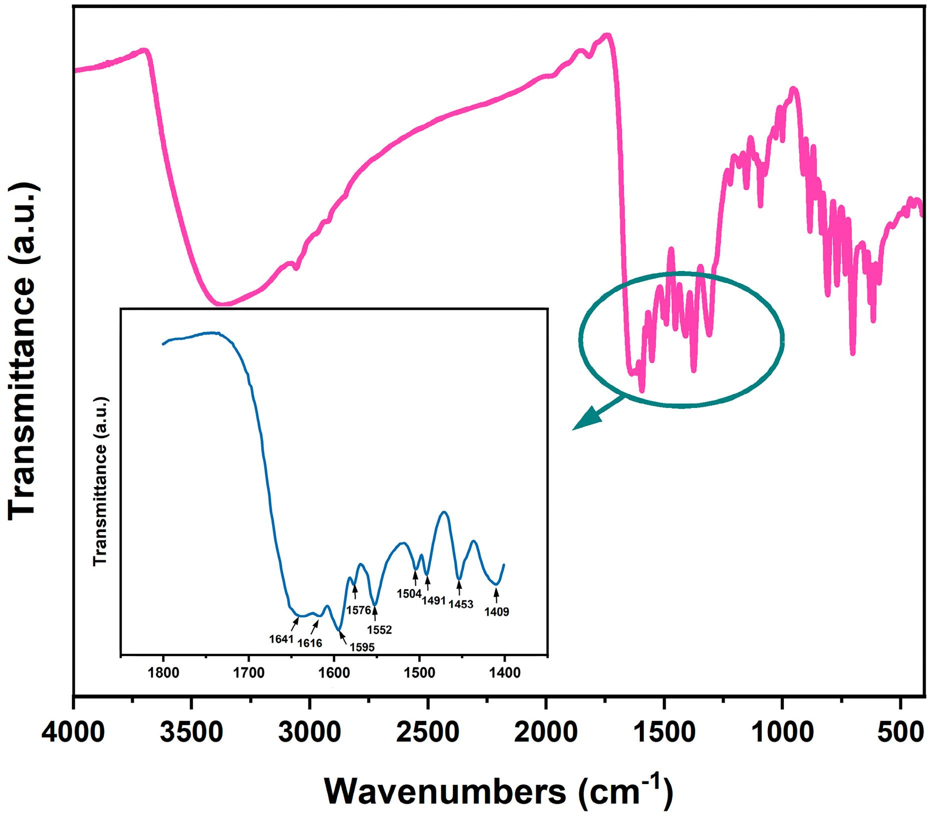

Figure S19. FT-IR spectrum of $[Gd(DPPDA)_2](DIPEA)$ in the range of 4000~400 cm^{-1} (The illustration is enlarged FT-IR spectrum in the range of 1800~1400 cm^{-1}).
